# Demographic characteristics and mental health condition of Tehran Municipality employees during the COVID-19 pandemic

**DOI:** 10.1186/s12879-024-09181-8

**Published:** 2024-03-06

**Authors:** Elham Kalhori, Nahid Khodakarami, Mostafa Hamdieh, Roya Gholami, Sareh Dashti

**Affiliations:** 1 Men’s Health and Reproductive Health Research Center, Shahid Beheshti University of Medical Sciences, Tehran, Iran; 2 Department of Psychosomatic Medicine, Taleghani Hospital, Shahid Beheshti University of Medical Sciences, Tehran, Iran; 3 Department of Midwifery, Faculty of Nursing and Midwifery, Tehran Medical sciences, Islamic Azad university, Tehran, Iran; 4 Department of Midwifery, Faculty of Nursing and Midwifery, Mashhad Medical Sciences, Islamic Azad University, Mashhad, Iran; 5 Department of Public Health, Faculty of Paramedicine, Mashhad Medical sciences, Islamic Azad University, Mashhad, Iran

**Keywords:** COVID-19, Employees, Health, Mental health, Demographic characteristics

## Abstract

**Background:**

Coronavirus disease 2019 (COVID-19) has become a global health issue with various effects on the physical and mental state of the societies. The aim of this study was to identify the demographic characteristics and mental health condition of Tehran Municipality employees during the COVID-19 pandemic.

**Methods:**

This cross-sectional study was performed on Tehran Municipality employees in 2020–2021. Participants were selected using stratified random sampling and were divided into COVID-19 and uninfected groups. Demographic characteristics, COVID-19 risk behaviors, General Health Questionnaire-28 (GHQ-28), and Well- Being Social Inventory were filled for all participants.

**Results:**

A total of 510 participants (363 uninfected participants and 147 participants with COVID-19) were evaluated. The prevalence of female gender was significantly higher in COVID-19 group compared to uninfected group (*p* < 0.001). There was a significant difference between groups in terms of education level (*p* < 0.001), prevalence of excess weight (*p* < 0.001), and working sector (*p* < 0.001). The uninfected group mainly had low contact with clients (*p* < 0.001) and few underlying diseases (*p* = 0.004) compared to the COVID-19 group. The mean GHQ-28 and Well- Being Social Inventory were significantly higher in the uninfected group compared to the COVID-19 group (*p* = 0.002 and *p* < 0.001, respectively). The prevalence of no and low contact level was significantly higher in the high infection cluster compared to moderate and low infection clusters (*p* = 0.024).

**Conclusions:**

The findings of this study indicated that all workers should be educated about the significance of social distancing and follow the recommendations regardless of their level of contact with clients.

## Background

The coronavirus disease 2019 is caused by a new strain of the coronavirus family namely severe acute respiratory syndrome coronavirus 2 (SARS-Cov-2) [1]. Nearly four months after the first report of the disease, the global spread of COVID-19 made the World Health Organization (WHO) declare it a pandemic [2]. On February 19, 2020, the Ministry of Health and Medical Education of Iran announced the first definitive case of COVID-19 [3]. Within 100 days from the identification of the first case, the number of COVID-19 patients reached 220,000 and the COVID-19 related mortality reached 10,000 [4].

COVID-19 can be transmitted through inhaled droplets that are produced through cough or breathing of an infected person as well as contact with contaminated surfaces [5]. Due to the mode of transmission of COVID-19, the most important factor in breaking the virus transmission chain is the preventive behaviors of the general public and social distancing [6–8]. Therefore, a global movement was shaped to enforce movement restriction and public education through national media and social networks [9, 10]. In order to reduce disease transition transmission hot spots should be identified and proper interventions should be designed to reduce contamination in these regions [11–13].

The burden of COVID-19 complications is growing with time and new aspects are identified each year. One of the complications related to COCID-19 is the mental effects due to the disease and the social distancing related to the contamination management strategies [14–16]. The mental health complications of COVID-19 may not only affect the health of individuals, but may also affect disease transmission through the effects of mental health condition on performing risk behaviors and preventive measures [17, 18].

In Iran, a movement restriction order was implemented from early March 2020 with the exception of workers in some critical occupations, other occupations were closed or restricted. City municipal employees were among the employees that did not stop services during the pandemic. Considering the variation in the education level, job description and working place, it was hypothesized that the distribution of infected workers may represent the COVID-19 transmission hotspots in the city. The aim of this study was to identify the demographic characteristics and mental health condition of Tehran Municipality employees during the COVID-19 pandemic.

## Methods

### Study participants

This cross-sectional study was performed on all registered employees of the Tehran Municipality in all city districts affiliated to the municipality in 2020–2021. The total number of employees was estimated to be approximately 60,000.

Sample size of the present study was calculated using the Morgan table and Cochran’s formula for uninfected and COVID-19 participants [19]. The sample size for uninfected and participants with COVID-19 were 386 and 200 individuals, respectively. Healthy participants were selected based on stratified random sampling. Sampling was performed in two steps. In the first step strata were defined based on the percentage of employees in each rank (constituencies, deputies, and supervised organizations). In the second step, employees from the city districts were systematically selected based on their population proportion from the list of employees in each stratum (Table 1). The study protocol was approved by the Tehran Urban Planning and Research Center ethical committee the number of the approval is: 383,389. participants gave informed consent before taking part.

**Table 1:** Distribution of the strata in the city municipality and the selected study participants

Stratum	Population size (N)	Stratum proportion (%)	Sample size (n)
Secretariat	251	0.42%	1
General office of the Mayor	5286	9.0%	34
Deputy of Finance	4352	8.0%	30
Human recourse development	1194	2.0%	8
Deputy of planning	597	1.0%	3
Urban planning and architecture	748	1.0%	3
Transportation and traffic	16,363	27.0%	104
City services	8355	14.0%	54
Social and cultural affairs	2211	4.0%	15
Technical and civil	1396	2.0%	8
Regional affairs	55	0.09%	1
Municipality of regions	18,897	32.0%	123
Total	59,705	100%	386

Participants with COVID-19 were selected from the latest list of the infected individuals obtained from the Tehran Municipality Health Center and the Tehran Deputy of Health based on global sampling from 20 January 2021 to 20 February 2021. The inclusion criteria for participants with COVID-19 were being diagnosed with COVID-19 based on RT-PCR test within the past week. Exclusion criteria for participants with COVID-19 were maximum of two months elapsed since the onset of the disease and having history of mental disorders.

After determining the names and details of the individuals in the list of employees of each stratum, administrative permits were obtained to access the contact number of the participants. Data collection was performed using interviews. The interviewers were experts who were briefed about filling the questionnaires in a two-hour session.

### Study instruments

The study questionnaire included demographic data (age, gender, living address, education level, monthly income, type of housing, house surface area, and household size), participants were also asked about their weight, level of contact with clients, past medical and mental history, and extent of following the social distancing and preventive guidelines in the form of self-report, as well as the number of relatives and close friends with COVID-19 and number of deceased relatives or friends due to COVID-19. The Well- Being Social Inventory and General Health Questionnaire-28 (GHQ-28) questionnaires were also filled for the participants.

The demographic and self-report questionnaires were evaluated by an expert panel to approve the construct and content validity. The expert panel including two experts in Reproductive Health, one Medical Doctor with more than 10 years’ experience in community health, and one expert in midwifery with previous experience in community health services. The questionnaire was reevaluated after piloting. The Well- Being Social Inventory questionnaire is a 33-item questionnaire. The questionnaire was previously validated in Iran and the Cronbach’s alpha for the questionnaire was reported to be 0.83 [20]. The GHQ-28 questionnaire is a 28-item questionnaire that is scored based on a four-point Likert scale. The GHQ-28 scores may range between 0 and 84. The questionnaire was previously validated in Iran. The sensitivity, specificity and misclassification rate of the questionnaire was reported to be 87.4%, 93.8% and 8.2%, respectively and the concurrent validity of the questionnaire was approved against Symptom Checklist-90-Revosed questionnaire [21].

Due to the pandemic restrictions, an online survey was conducted and all the participants filled the questionnaires online. This method could provide the opportunity to obtain data from employees who were in sick leave at the time of data collection.

### Study procedure

Before completing the questionnaires, participants were briefed regarding the study, its purpose, and the anonymity of data analysis. Participants were then asked to sign an informed consent. Then, the questionnaires were filled for all participants (healthy and COVID-19 groups) during an interview. Filling the questionnaires took approximately 25 min for each participant. After excluding participants based on exclusion criteria 147 participants in the COVID-19 and 363 participants in the healthy group were evaluated.

## Results

In this study 363 uninfected participants and 147 participants with COVID-19 were evaluated. The age of the participants ranged between 20 and 53 years old. Comparison of the demographic characteristics between healthy and COVID-19 groups are presented in Table 2. Majority of the COVID-19 participants were female which was significantly higher compared to the gender distribution in the healthy group (*p* < 0.001). There was a significant difference between groups in terms of education level indicating that more participants in the COVID-19 group had Master’s degree, while more participants in the healthy group had Bachelor’s degree (*p* < 0.001). The prevalence of excess weight was significantly higher in participants in the COVID-19 group compared to the uninfected group (*p* < 0.001). In terms of working sector, participants in the COVID-19 group were more occupied in secretariat, deputy of finance, human resource and development, urban planning and architecture, city services, social and cultural affairs, technical and civil, and regional affairs sectors compared to the uninfected group who were more occupied in general office of the mayor, deputy of planning, transportation and traffic, and municipality regions (*p* < 0.001).

**Table 2:** Comparison of demographic characteristics of the study participants between healthy and COVID-19 groups

Variable		COVID-19 group *n* = 147	Healthy group *n* = 363	p
Gender	Female	103 (70.1%)	126 (34.7%)	< 0.001*
	Male	44 (29.9%)	237 (65.3%)	
Education level	Illiterate or primary education	1 (0.7%)	1 (0.3%)	< 0.001*
	Highschool and below	9 (6.1%)	4 (1.1%)	
	High school graduate	45 (30.6%)	27 (7.4%)	
	Diploma	11 (7.5%)	31 (8.5%)	
	Bachelor	0 (0.0%)	173 (47.7%)	
	Masters	78 (53.1%)	110 (30.3%)	
	PhD and above	3 (2.0%)	15 (4.1%)	
	Clerical education	0 (0.0%)	2 (0.6%)	
Age	20–30 years old	10 (6.8%)	26 (7.2%)	0.931
	30–40 years old	60 (40.8%)	153 (42.1%)	
	40 years old and above	77 (52.4%)	184 (50.7%)	
Marital status	Single	23 (15.6%)	69 (19.0%)	0.163
	Married	119 (81.0%)	291 (80.2%)	
	Widowed	2 (1.4%)	1 (0.3%)	
	Divorced	3 (2.0%)	2 (0.6%)	
Weight (kg)	40–60 kg	8 (5.4%)	36 (9.9%)	< 0.001*
	60–90 kg	65 (44.2%)	245 (67.5%)	
	90 kg and above	74 (50.3%)	82 (22.6%)	
Working sector	Secretariat	12 (8.2%)	1 (0.6%)	< 0.001*
	General office of the Mayor	9 (6.1%)	40 (11.0%)	
	Deputy of Finance	14 (9.5%)	10 (2.8%)	
	Human recourse development	13 (8.8%)	19 (5.2%)	
	Deputy of planning	1 (0.7%)	6 (1.7%)	
	Urban planning and architecture	2 (1.4%)	4 (1.1%)	
	Transportation and traffic	22 (15.0%)	70 (19.3%)	
	City services	30 (20.4%)	59 (16.3%)	
	Social and cultural affairs	11 (7.5%)	15 (4.1%)	
	Technical and civil	5 (3.4%)	11 (3.0%)	
	Regional affairs	5 (3.4%)	2 (0.6%)	
	Municipality of regions	23 (15.6%)	125 (34.4%)	
Living place condition	Good	67 (45.6%)	120 (35.9%)	0.109
	Moderate	68 (46.3%)	189 (56.6%)	
	Poor	12 (8.2%)	25 (7.5%)	

* Significant difference in distribution pattern using the chi-square test

**Fig. 1:**
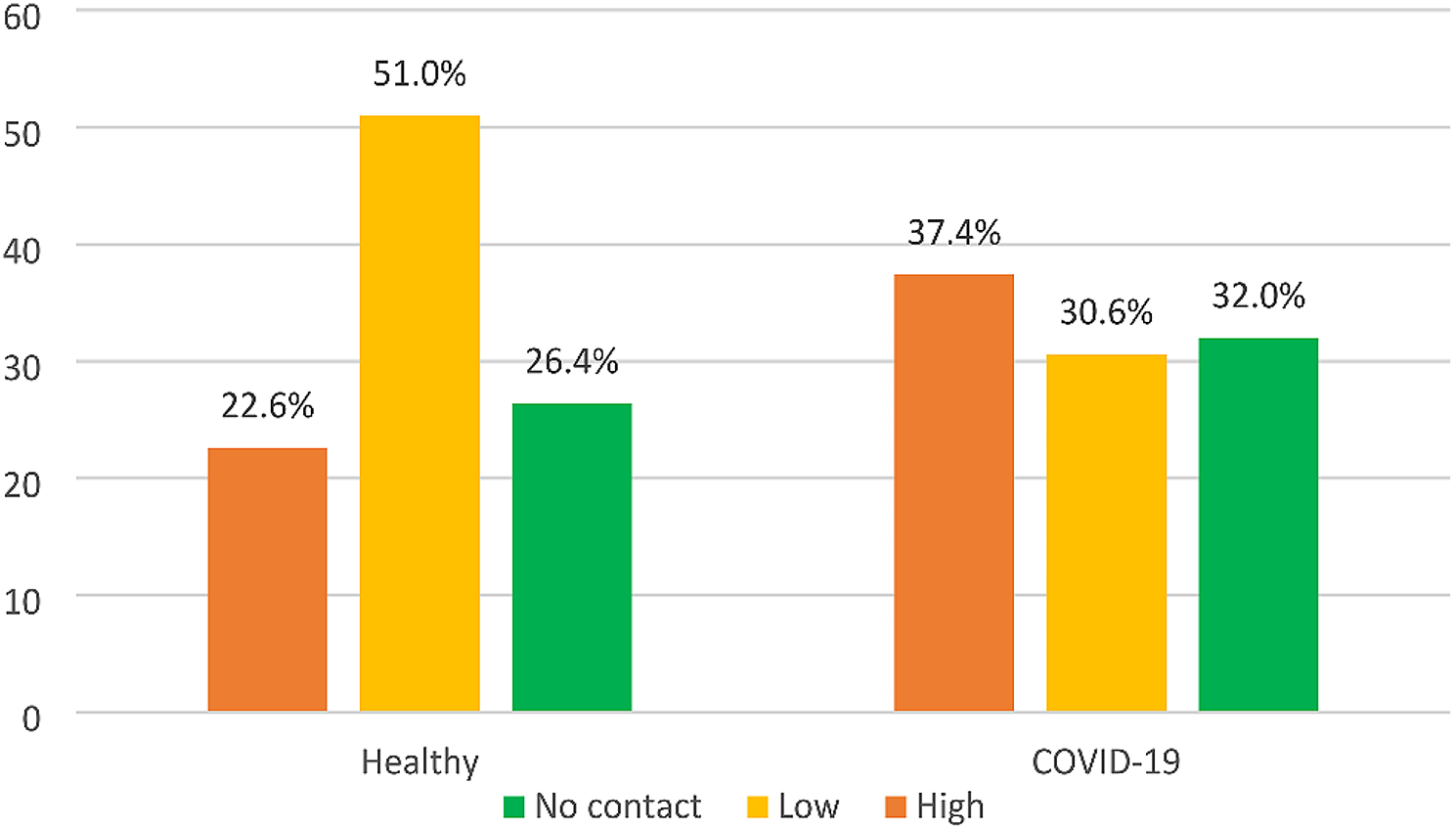
Comparison of the distribution pattern of the level of contact with client between study groups

Comparison of the level of contact with clients between COVID-19 and uninfected groups are presented in Fig. 1. There was a significant difference in the distribution pattern of the level of contact and study groups indicating that the uninfected group mainly had low contact with clients compared to the COVID-19 group (*p* < 0.001).

**Fig. 2:**
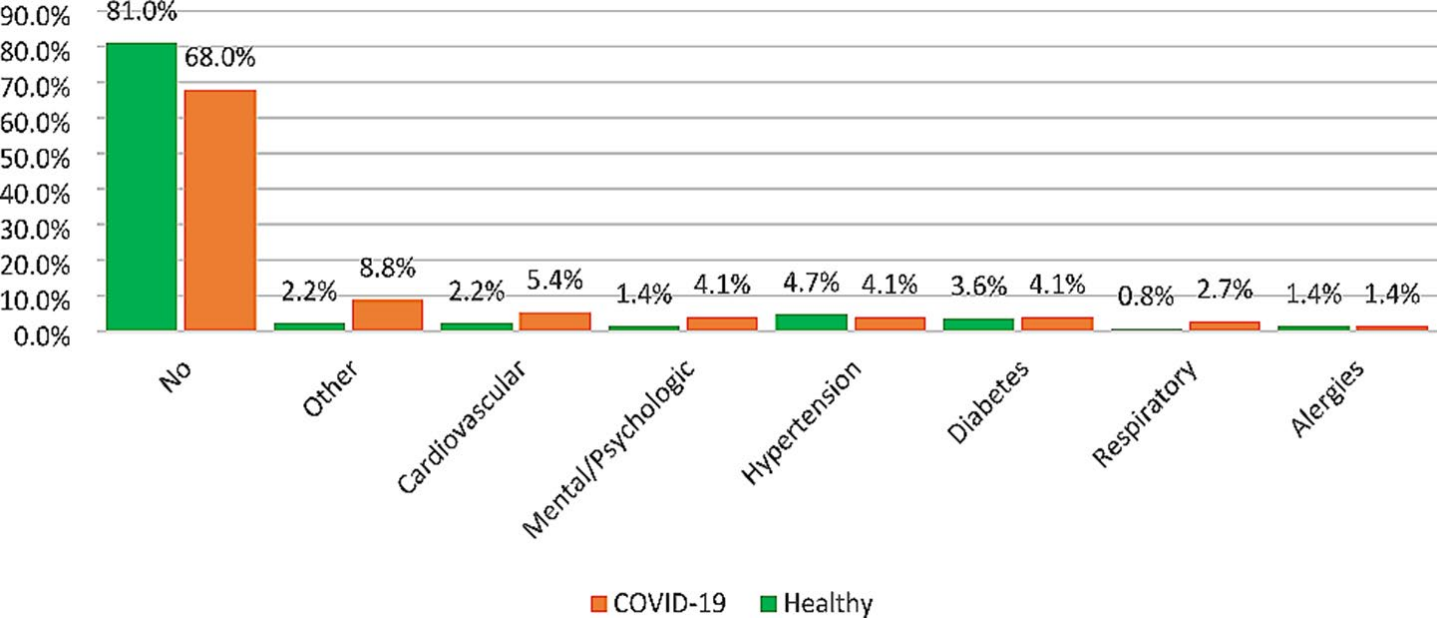
Comparison of the distribution pattern of underlying diseases between study groups

Comparison of the distribution pattern of underlying diseases between the COVID-19 and uninfected groups is presented in Fig. 2. There was a significant difference between groups in terms of the distribution pattern of underlying diseases indicating that the prevalence of any underlying diseases was significantly lower in the uninfected participants compared to participants with COVID-19 (*p* = 0.004).

City districts were clustered into three groups, low, moderate, and high infection rate based on the percentage of participants with COVID-19 from the study population (Table 3). The lowest infection rates were observed in districts 22, 19 and 21, while the highest infection rates were observed in districts 11, 16, and 8.

**Table 3:** Clusters of COVID-19 infection rates based on the study population

Cluster	District	Infection rate (%)
Low	22	65.00%
	19	63.60%
	21	60.00%
	20	57.10%
	4	55.80%
	15	50.00%
Moderate	5	65.40%
	1	66.70%
	13	66.70%
	17	66.70%
	23	66.70%
	10	75.00%
	12	75.00%
	2	75.00%
	9	77.80%
	7	78.60%
	14	79.50%
	18	80.00%
High	6	84.20%
	3	85.70%
	8	88.20%
	16	88.20%
	11	93.80%

Among the COVID-19 and uninfected groups, the prevalence of positive history for mental or psychological disorders before COVID-19 pandemic were 7(4.8%) and 6 (1.7%), respectively, while during the COVID-19 pandemic the prevalence of mental or psychological disorder increased in COVID-19 and uninfected groups to 8 (5.4%) and 16 (4.4%), respectively. There was no significant difference in the prevalence of mental or psychological disorders between groups before (*p* = 0.066) and after pandemic (*p* = 0.250).

The mean ± SD score for the GHQ-28 in the COVID-19 and uninfected groups were 27.98 ± 0.24 and 28.72 ± 0.12, respectively, while the mean score of the Well- Being Social Inventory in COVID-19 and uninfected groups were 14.02 ± 0.34 and 20.91 ± 0.21, respectively. The mean GHQ-28 and Well- Being Social Inventory were significantly higher in the uninfected group compared to the COVID-19 group (*p* = 0.002 and *p* < 0.001, respectively).

Comparison of study variables between clusters are presented in Table 4. There was only a significant difference in the level of contact with clients between the infection clusters indicating that the prevalence of no and low contact level was significantly higher in the high infection cluster compared to moderate and low infection clusters (*p* = 0.024). There was no significant difference in other risk behaviors between clusters.

**Table 4:** Comparison of the study variables between clusters of infection rate

Variable		Low Frequency (%)	Moderate Frequency (%)	High Frequency (%)	p
Level of contact with clients	No	25 (24.3%)	86 (28.0%)	30 (32.3%)	0.024*
	Low	39 (37.9%)	143 (46.6%)	47 (50.5%)	
	High	39 (37.9%)	78 (25.4%)	16 (17.2%)	
Types of gatherings participated during the past two months	Ceremony	2 (2.0%)	11 (3.6%)	1 (1.1%)	0.052
	Religious	2 (2.0%)	0 (0.0%)	2 (2.0%)	
	Clinic/Hospital/Pharmacy	1 (1.0%)	15 (5.0%)	2 (2.2%)	
	Public transportation	2 (2.0%)	6 (2.0%)	1 (1.1%)	
	Work place	49 (48.5%)	122 (40.3%)	38 (41.8%)	
	Travel	6 (5.9%)	3 (1.0%)	1 (1.1%)	
	Shopping/Social places	1 (1.0%)	6 (2.0%)	2 (2.2%)	
	Two or more of the above	38 (37.6%)	137 (45.2%)	43 (47.3%)	
	Other	0 (0.0%)	3 (1.0%)	1 (1.1%)	
Mask usage in public/work place	Never	0 (0.0%)	0 (0.0%)	0 (0.0%)	0.226
	< 25% of the times	0 (0.0%)	2 (0.7%)	0 (0.0%)	
	25–50% of the times	3 (2.9%)	1 (0.3%)	0 (0.0%)	
	50–75% of the times	4 (3.9%)	21 (6.8%)	4 (4.3%)	
	> 75% of the times	96 (93.2%)	283 (92.2%)	89 (95.7%)	
Hand washing in public/work place during the past two months	Never	0 (0.0%)	1 (0.3%)	1 (1.1%)	0.391
	< 25% of the times	2 (1.9%)	1 (0.3%)	0 (0.0%)	
	25–50% of the times	11 (10.7%)	22 (7.2%)	4 (4.3%)	
	50–75% of the times	17 (16.5%)	54 (17.6%)	15 (16.3%)	
	> 75% of the times	73 (70.9%)	229 (74.6%)	72 (78.2%)	
Social distancing in public/workplace during the past two months	Never	4 (3.9%)	5 (1.6%)	0 (0.0%)	0.427
	< 25% of the times	5 (4.9%)	9 (2.9%)	1 (1.1%)	
	25–50% of the times	12 (11.7%)	26 (8.5%)	8 (8.7%)	
	50–75% of the times	17 (16.5%)	65 (21.2%)	19 (20.7%)	
	> 75% of the times	65 (63.1%)	202 (65.8%)	64 (69.6%)	
Use of shield in public/workplace during the past two months	Never	97 (94.2%)	271 (88.6%)	87 (94.6%)	0.097
	< 25% of the times	0 (0.0%)	17 (5.6%)	3 (3.3%)	
	25–50% of the times	2 (1.9%)	8 (2.6%)	0 (0.0%)	
	50–75% of the times	0 (0.0%)	0 (0.0%)	0 (0.0%)	
	> 75% of the times	4 (3.9%)	10 (3.3%)	2 (2.2%)	
Use of disinfectants in public/workplace during the past two months	Never	1 (1.0%)	4 (1.3%)	0 (0.0%)	0.726
	< 25% of the times	1 (1.0%)	3 (1.0%)	3 (3.3%)	
	25–50% of the times	6 (5.9%)	13 (4.2%)	2 (2.2%)	
	50–75% of the times	14 (13.9%)	40 (13.1%)	11 (12.1%)	
	> 75% of the times	79 (78.2%)	246 (80.4%)	75 (82.4%)	

* Significant difference in distribution pattern using the chi-square test

## Discussion

The results of the present study confirmed the hypothesis that COVID-19 had impacted the mental well-being of municipal employees. This finding was consistent with the results of previous studies [22, 23]. From the perspective of social psychologists, psychological well-being is feeling of satisfaction, which is defined as well-being, happiness, and achieving full individual potentials, and includes life satisfaction, positive and negative emotions [24]. The structure of psychological well-being is defined as the development of individual’s true talents and includes six components: purposeful life, positive relationship with others, personal growth, self-acceptance, independence or autonomy, and mastery of the environment [25]. Anxiety caused by COVID-19 itself is an acute event that can disrupt the psychological well-being of individual and society. Studies have shown a significant relationship between stress, anxiety and depression and psychological well-being. Severe anxiety may negatively lead to high risk of mortality [26].

According to the findings of the present study, the higher the rate of personal growth and positive communication with others, the lower the level of COVID-19 related anxiety. Personal growth means having a feeling of continuous growth and high capacity and a sense of increasing efficiency and knowledge [27]. There is a vicious circle between anxiety and psychological well-being, so that enduring anxiety endangers individual’s psychological well-being and thus leads to anxiety in different situations [28]. According to the findings of this study, responsibility, as another component of health promotion lifestyle, was inversely related to COVID-19 related anxiety. It seems that responsibility makes a person sensitive and responsible for maintaining and promoting his health. Physicians who have high health responsibilities usually are cautious about their health and report less anxiety [29]. Health requires following a healthy lifestyle, as a result, people need to learn strategies to deal with anxiety [30, 31]. Furthermore, stress and anxiety may suppress immune system and increase the risk of acquiring COVID-19 [32].

The results of the present study indicated that the personal and spiritual growth of the participants, their relationships with others, and health responsibility were inversely related to anxiety in acute situations such as COVID-19 pandemic. The results of the present study showed that the incidence of COVID-19 was higher in older participants compared to younger participants. This finding might be related to the higher rate of underlying and chronic diseases in this age group [33]. In this regard, the results of the present study showed that three factors including male gender, hypertension and hemoglobin less than 10 were the predictors for hospitalization in COVID-19. This finding was similar to the findings of previous studies [34, 35].

According to the present study, among the demographic and habitat variables, there was only a significant relationship between the level of contact with the client and the disease clusters. In this study, participants living in high-incidence clusters had lower levels of contact with the client. A 2020 study by Norworth et al. In Germany of 87 nurses, 22 physicians, and 18 hospital staff during the first wave of globalization of Covid 19 reported that participants’ use of masks and hand hygiene Who were active in the Covid 19 divisions were significantly higher than the participants who were active in the non-Covid divisions. The authors of this article attributed the reason for the higher level of protection protocols in the participants who worked in Covid 19 wards to the higher awareness of this group about the use of protection protocols due to the type of daily activity (Norworth, Matner, Auschwitz and et al., 2020). Another study conducted by Kim et al. In 2021 on 2884 health care workers in 6 countries: England, Germany, France, Italy, Spain and the United States showed that the level of access to protective equipment as well as the degree of close contact with Patients were directly related to the rate of staff infection as well as the duration of their illness (if any) (Kim, Hedge, Lafiera et al., 2021). Based on the findings of these two studies, the findings of the current study can be justified as follows. More access to protective equipment, as well as the knowledge and practice of participants who had more business contact with the client, probably protected them against Covid 19. Because they were aware of the high risk of getting Covid 19 at work, they followed health protocols more and better. It can also be inferred that by providing protective equipment to employees who have more contact with the client, easier access to these devices has protected them against Covid 19 infection. However, the lack of a significant correlation between the use of masks and other protective devices between clusters does not support these theories. Given that the questionnaire was self-reported and the participants had declared the status of using the mask as well as their infection without documents, it is possible that the results of this study were biased in assessing the observance of health protocols. Also, the difference in the number of participants in the study groups and the small number of participants in some of the groupings, has reduced the validity of the statistical test. On the other hand, according to a study by Kim et al., In addition to occupational exposure, exposure in the living environment and outside the workplace can also have a transient effect on a person.

One of the limitations of this study was clustering of infection zones based on the infection of employees; therefore, it cannot be said with certainty that the degree of infection or contamination of the municipality areas were consistent with this clustering. Another limitation of this study was related to the inclusion of city council employees, which does not include children and elderly population and unemployed citizens. Therefore, the findings of this study may not be generalizable to all groups of citizens. However, due to the gender distribution and the diversity in the level of education and occupations, the findings of this study can still be used as a guide in determining the demographic and mental health risk factors for COVID-19.

## Conclusions

The findings of this study indicated that the behavioral risk factors for COVID-19 among Tehran Municipality employees were acceptable and were not related to the incidence of COVID-19. Therefore, it can be said that the fear of acquiring the disease from workplace had forced the employees with high level of contact with clients to follow the recommendations. However, employees with less client contact were less likely to follow prevention strategies. Therefore, employees should be educated regarding the roots or transmission of diseases regardless of their working sector and level of contact with clients.

## What is already known about this subject?

- COVID-19 has made a great global health and economic burden.
- Disease prevention requires the understanding of predisposing factors especially in services that may not be closed.

## What this study adds

- Occupational contact level plays an important role in contamination.
- Regular mask mandate and social distancing rules might need to be revised in high contact jobs.

### What impact this may have on practice or policy

- Monitoring COVID-19 and other contagious diseases in high importance jobs are required to identify high occupational posts.
- Social distancing and mandates should be individualized based on job description and contact level,

## Data Availability

The datasets generated and analysed during the current study are not publicly available due but are available from the corresponding author on reasonable request.
